# The influence of different aspects of grouse moorland management on nontarget bird assemblages

**DOI:** 10.1002/ece3.5613

**Published:** 2019-09-26

**Authors:** Nick A. Littlewood, Tom H. E. Mason, Martin Hughes, Rob Jaques, Mark J. Whittingham, Stephen G. Willis

**Affiliations:** ^1^ School of Natural and Environmental Sciences (SNES) Newcastle University Newcastle‐Upon‐Tyne UK; ^2^ Conservation Ecology Group Department of Biosciences Durham University Durham UK

**Keywords:** burning, conservation conflict, curlew, golden plover, human‐wildlife conflict, predator control, red grouse, snipe

## Abstract

1. Conflict between stakeholders with opposing interests can hamper biodiversity conservation. When conflicts become entrenched, evidence from applied ecology can reveal new ways forward for their management. In particular, where disagreement exists over the efficacy or ethics of management actions, research clarifying the uncertain impacts of management on wildlife can move debates forwards to conciliation.
2. Here, we explore a case‐study of entrenched conflict where uncertainty exists over the impacts of multiple management actions: namely, moorlands managed for the shooting of red grouse (willow ptarmigan) *Lagopus lagopus* in the United Kingdom (UK). Debate over how UK moorlands should be managed is increasingly polarized. We evaluate, for the first time at a regional scale, the relative impacts of two major moorland management practices—predator control and heather burning—on nontarget bird species of conservation concern.
3. Birds were surveyed on 18 estates across Northern England and Southeast Scotland. Sites ranged from intensively managed grouse moors to moorland sites with no management for grouse shooting. We hypothesised that both targeted predator control and burning regimes would enhance ground‐nesting wader numbers and, as a consequence of this, and of increased grouse numbers, nontarget avian predators should also be more abundant on heavily managed sites.
4. There were positive associations between predator control and the abundance of the three most widespread species of ground‐nesting wader: strong effects for European golden plover *Pluvialis apricaria* and Eurasian curlew *Numenius arquata* and, less strongly, for common snipe *Gallinago gallinago*. These effects saturated at low levels of predator control. Evidence for effects of burning was much weaker. We found no evidence of enhanced numbers of nontarget predators on heavily managed sites.

## Graphical abstract

On upland sites in Northern England and southern Scotland, populations of some bird species of conservation concern, namely golden plover, curlew, and snipe, were driven more by increasing effort spent on legal predator control than by rotational heather burning. There was saturating effect of predator control, such that populations of these waders reached a plateau above which additional predator control effort added little benefit. Large predatory birds were not detected more on sites where legal predator control effort was high, despite higher availability of potential food resources.

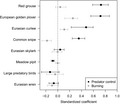

## 1 INTRODUCTION

Biodiversity conservation often produces tension between bodies wishing to maintain species of conservation concern and those that wish to use the areas occupied by species for other purposes (Sillero‐Zubiri, Sukumar, & Treves, 2007). Well publicized examples of such conflicts of interest include the reintroduction or recovery of large predators (e.g., gray wolves *Canis lupus* in Europe and the USA—Mech, 2017), the control of species that limit agricultural productivity (e.g., geese and common crane *Grus grus* on European farmland—Mason, Keane, Redpath, & Bunnefeld, 2017), and the occurrence of threatened populations in areas of prime real estate development (e.g., the clearance of coastal habitats for development—Drius et al., 2019) or of high extractive use value (e.g., spotted owls *Strix occidentalis* in old growth forests—Wan, Ganey, Vojta, & Cushman, 2018). Such conflicts of interest can become entrenched into opposing factions, with little opportunity to realize solutions (Thirgood & Redpath, 2008). Resolving these relies not only on understanding the human elements of conflicts (Dickman, 2010), but also the processes through which humans and wildlife interact (Redpath & Sutherland, 2015). In particular, disagreements over the effects or ethics of management interventions on wildlife can be a key driver of conflict and applied ecological studies that clarify these effects can provide important evidence for moving these debates forward (Redpath & Sutherland, 2015).

Entrenched, long‐running conflict exists in the United Kingdom (UK) uplands between stakeholders favoring the sport shooting of red grouse and those opposing it (Sotherton, Tapper, & Smith, 2009; Thompson, Amar, Hoccom, Knott, & Wilson, 2009). Previous applied ecological research has informed arguments surrounding this conflict by revealing that: (a) illegal killing of raptors occurs on some grouse moors (e.g., Murgatroyd et al., 2019); and (b) predation by raptors can make driven grouse shooting economically unviable (Thirgood & Redpath, 2008). More recently, debate around environmental issues associated with driven grouse shooting has broadened to include wider environmental impacts of moorland management, such as ecosystem service delivery through carbon storage and flood alleviation (e.g., Sotherton, Baines, & Aebischer, 2017; Thompson et al., 2016). However, the way in which moorland management, aimed at maximizing grouse numbers, impacts on wider ecological assemblages is still contested. Some studies have demonstrated that management for grouse shooting can have a positive impact on certain upland species of conservation interest (see Table 1). One or both of two major management activities on such moorland, vegetation burning and predator control, are thought to be the primary driver of increasing densities of some nontarget bird species. However, the individual impacts of these management activities on nontarget species remain to be fully resolved.

**Table 1:** Key studies considering the effects of grouse moor management on upland bird assemblages in the UK

Reference	Background	Number of sites	Location	Higher numbers associated with grouse moor management	Lower numbers associated with grouse moor management
Baines et al. (2008)	Reduction, then cessation of grouse moor management	One site	Southwest Scotland	European golden plover, northern lapwing, Eurasian curlew, red grouse, Eurasian skylark^a^	Carrion crow, common snipe
Buchanan et al. (2017)	Surveys of plots across a range of sites selected on basis of vegetation cover	159 plots (each 2 km^2^)	North Pennines, South Pennines, southern Scotland and Wales	Red grouse, Eurasian curlew, European golden plover	
Fletcher et al. (2010)	Experimental manipulation of predator numbers	Four plots (6.1–6.9 km apart)	Northumberland	Northern lapwing, European golden plover, Eurasian curlew, red grouse^b^	
Newey et al. (2016)	Field surveys of sites reporting a range of management priorities	26 landholdings	Scotland	Eurasian curlew, common buzzard, short‐eared owl, black‐headed gull, common sandpiper^c^	Black grouse, merlin, northern raven, ring ouzel, meadow pipit, Eurasian skylark, northern wheatear, carrion/hooded crow^c^
Tharme et al. (2001)	Surveys across grouse moors and other upland estates	320 squares (each 1 km^2^) on 122 estates	Eastern Scotland and Northern England	European golden plover, northern lapwing, red grouse, Eurasian curlew	Meadow pipit, Eurasian skylark, whinchat, carrion/hooded crow

a Hen harrier was more abundant following cessation of illegal control, but then declined following complete cessation of grouse moor management.

b Meadow pipits also showed increased breeding success associated with predator control, but not a subsequent increase in numbers.

c Three different ordination methods were used in the analysis. Species named in the table are those where the association (negative or positive) with grouse shooting was significant (*p* < .05) in at least one of these.

The most intensive form of grouse shooting, driven grouse shooting, in which birds are flushed toward lines of concealed shooters, takes place over approximately 3,700 km^2^ of UK moorlands. This equates to about 15% of the UK's moorland area, with particular concentrations in Northern England and parts of eastern Scotland (Redpath, Amar, Smith, Thompson, & Thirgood, 2010). The dominant dwarf shrub, heather (*Calluna vulgaris*), is burned in patches to produce differently aged stands that provide food and shelter for red grouse. Legal predator control entails removing populations or reducing abundances of some birds and mammals that might predate red grouse eggs, chicks, or adults. These two management interventions create habitat conditions, and a low predator environment, that may influence populations of other moorland bird species. In particular, burning may facilitate foraging opportunities for waders among the resultant short vegetation while predator control is likely to reduce losses of eggs and chicks, and, to a lesser extent, adult birds.

An internationally important bird assemblage breeds on unenclosed moorlands in the UK, including several species considered to be among the UK's highest conservation priorities (Eaton et al., 2016). Moorlands hold a substantial proportion of the UK's breeding Eurasian curlews, which constitute approximately 60% of the entire EU population. This species is listed as Near Threatened in a global context (BirdLife International, 2017) and considered to be the UK's highest bird conservation priority (Brown et al., 2015). Populations of commoner species include what are possibly the highest densities of Eurasian skylark *Alauda arvensis* and meadow pipit *Anthus pratensis* globally (Thompson, Macdonald, Marsden, & Galbraith, 1995), these being red‐ and amber‐listed species, respectively in the UK (Eaton et al., 2016). Thus, appropriate management of upland moorland is critical for preserving populations of key bird species.

Previous studies have generally shown a positive relationship between grouse moor management and populations of red grouse and of ground‐nesting waders. Among waders, such a relationship has been reported for European golden plover, northern lapwing *Vanellus vanellus* and Eurasian curlew (e.g., Baines, Redpath, Richardson, & Thirgood, 2008; Buchanan, Pearce‐Higgins, Douglas, & Grant, 2017; Newey et al., 2016; Tharme, Green, Baines, Bainbridge, & O'Brien, 2001; see Table 1) while studies focussing specifically on Eurasian curlew have also shown a positive correlation of abundance with gamekeeper activity (Douglas et al., 2014; Franks, Douglas, Gillings, & Pearce‐Higgins, 2017). In contrast, other species may be negatively associated with intensively managed grouse moors, including carrion/hooded crow *Corvus corone*/*cornix*, northern raven *Corvus corax*, Eurasian skylark, ring ouzel *Turdus torquatus*, northern wheatear *Oenanthe oenanthe*, and meadow pipit (e.g., Newey et al., 2016; Tharme et al., 2001). While crows are directly controlled on grouse moors, other species may be impacted by grouse moor management producing suboptimal habitat, relative to other upland areas that are not managed for sporting purposes. Additionally, illegal persecution is implicated as the major driver of suppressed numbers of some raptor species on moorlands managed for driven grouse shooting (e.g., Amar et al., 2012; Murgatroyd et al., 2019; Whitfield & Fielding, 2017).

Our study seeks to disaggregate the effects of moorland burning and predator control effort on upland breeding bird assemblages across a regional scale. Previous studies have not been able to address this directly as they have relied on proxy measures (such as crow abundance as an indicator of predator control effort) for one of these activities (Buchanan et al., 2017; Tharme et al., 2001). We consider absolute predator control effort so as to directly relate our findings back to management. Understanding predator control and burning effects is important for understanding the reliance of the wider bird assemblage on moorland management and the potential effects that change in these management actions may have on their national populations. This is an inherently challenging goal, as burning and predator control are usually carried out in the same areas, though experimental treatments at single sites provide some evidence for their individual impacts. For example, burning at one site with limited fox and crow control resulted in European golden plover numbers increasing (Douglas et al., 2017) while in another study (where conventional heather burning was maintained), predator control led to increases in breeding success, and subsequent increased breeding populations, of red grouse, northern lapwing, European golden plover, and Eurasian curlew, relative to areas without predator control (Fletcher, Aebischer, Baines, Foster, & Hoodless, 2010). Here, we investigate the impacts of these management actions across 104 survey plots, on 18 land holdings spread over an area spanning 133 km on its longest axis and varying in their intensity of burning and predator control. While these two activities are positively associated across our sites, we explored their relative support and effect sizes by fitting statistical models independently for each activity. We hypothesized that while some of the commoner upland species (Eurasian skylark, Eurasian wren *Troglodytes troglodytes*, and meadow pipit) would not be directly affected by either of these two management practices (albeit they may show positive or negative association with particular habitat features within upland estates), other nontarget species, including waders, such as European golden plover, Eurasian curlew, and common snipe, would strongly benefit from both management practices. We would expect red grouse, the target species of these management activities, also to benefit. Additionally, we tested the hypothesis that, with legal predator control, the consequent increased prey availability would support higher numbers of those bird species that could prey on grouse and wader species but which were legally protected.

## 2 METHODS

### 2.1 Study sites

**Figure 1:**
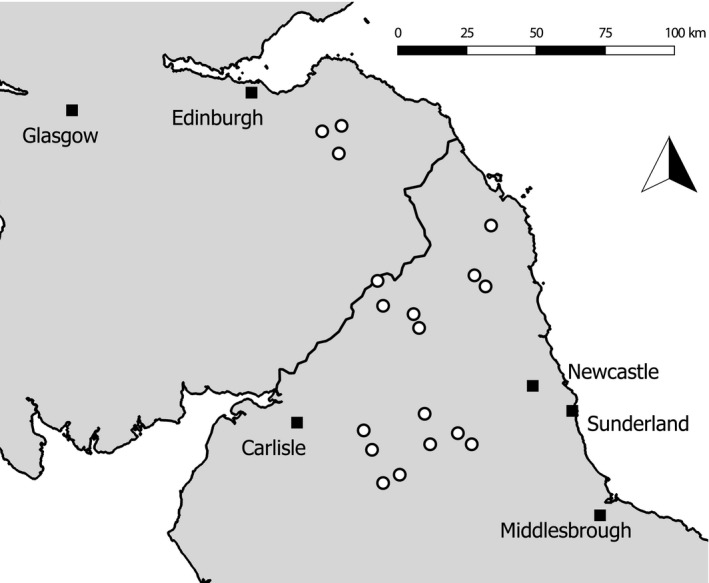
Location of the 18 estates surveyed in Northern England and southern Scotland and of large settlements in the region

Fieldwork was carried out in one hundred and four 1‐km^2^ survey squares, across 18 upland estates (mean estate size: 2,771 ha; *SE*: 433) in northern England and southern Scotland (Figure 1). These included 11 estates on which grouse shooting occurs, ranging from high intensity driven grouse shoots employing teams of gamekeepers to lower intensity sites on which no full‐time gamekeepers operate. The remaining estates were managed for a combination of conservation, livestock, and other sporting interests. Survey squares (1–12 per estate) were selected without prior knowledge of their bird assemblages. They were selected to encompass mainly heather‐dominated areas, and the number of squares selected was proportional to site size. Most were UK National Grid 1 km squares but ten were displaced to fit within estate boundaries. Survey squares were not located directly adjacent to each other, though some squares met at their corners, except for two instances, in each of which two squares shared a 500‐m border. Survey squares had a mean altitude ranging from 155 to 758 m and, while most were dominated by heather, some also contained areas of acid grassland.

### 2.2 Bird survey fieldwork

Birds were surveyed following Brown and Shepherd (1993) methodology. One fieldworker spent 80–100 min in a survey square, walking a route that enabled them to look in or over as much of the square as possible, using vantage points to scan for birds and using their judgment to maximize bird encounters across the square. Birds seen or heard were noted using standard species and breeding behavior codes. Field maps were annotated to record simultaneous registrations of birds of the same species, except for the very abundant meadow pipits, for which just the initial location of each new, or presumed new, bird was noted.

Each square was visited once between 15 April 2017 and 21 May 2017 and once between 23 May 2017 and 26 June 2017. Second visits to sites occurred at least 27 days after the initial visit (mean 36 days). This ensured that both early and late breeding species were recorded adequately. Fieldwork was carried out between 8.30 a.m. and 6.00 p.m., thus avoiding periods of rapidly changing bird activity levels in the early mornings and evenings. Surveys were not carried out when winds exceeded Beaufort force 5, in poor visibility or in rain exceeding light showers. Surveys were carried out by three field surveyors, with each square visited by a different surveyor on the two visits.

### 2.3 Interpreting field data

Estimates of the numbers of territories were generated for waders and for Eurasian wren. For Eurasian skylark, meadow pipit, large predatory birds (northern raven, owls and birds of prey excluding common kestrel *Falco tinnunculus*, and merlin *Falco columbarius*), and for red grouse, individual abundances were tallied, due to the high abundances of the former two former species and the large distances moved by grouse and the avian predators, making territory assessments unreliable.

Territory numbers for waders were estimated for each visit from breeding behaviors noted, following conventions of Brown and Shepherd (1993). Where it was not possible to determine whether birds were likely to be different individuals, a 500‐m cut‐off distance between map registrations was used to define territories (following Brown & Shepherd, 1993). The highest number of territories from the two visits was used as the estimate for that square (following Calladine, Garner, Wernham, & Thiel, 2009).

Territories of Eurasian wrens were determined similarly to waders except that single birds in suitable breeding habitat were also counted as representing a territory and a 200‐m threshold was used to identify different territories within a survey visit when this was not determined in the field. The same 200‐m threshold was used to establish whether birds noted on different visits belonged to the same territorial pair (following Calladine et al., 2009).

For red grouse, large predatory birds, Eurasian skylark and meadow pipit, simple indices of abundance were used. These indices summed counts of individuals seen of these species from both visits to a square. For red grouse, chicks were disregarded from counts. For meadow pipit and Eurasian skylark, counts were of adults and fledged juveniles (as these were typically difficult to differentiate from adults). For large predatory birds, no chicks or juveniles were observed during surveys.

### 2.4 Site management

Estate owners, tenants, agents, gamekeepers, and managers were interviewed to quantify the time spent on predator control activity on each of the 18 sites. These estimates primarily comprised activities of gamekeepers directly employed or contracted by the estates and were based on proportions of their time that were spent primarily on predator control. Estimates also included predator control carried out on estates by tenant graziers and by representatives from neighboring estates operating on the focal estate with consent. These data were converted into estimates of the number of full‐time equivalent staff exclusively carrying out predator control per 1,000 ha.

The area of each survey square under burning management was estimated from GoogleEarth images. These were accessed in August 2017 and comprised images spanning the years 2003–2016, with 84% of images being from 2007 onwards. An absence of signs of burning in aerial images is likely to indicate that a site has not been burned for 20 years or more (Yallop et al., 2006). While it was possible that burning may have been instigated on some areas since the images were taken, no substantial recent changes in burning management were reported during interviews with site contacts. An alternative, remotely sensed index of burning was also calculated. These estimates were calculated using remote‐sensing data (from Landsat 5 and 7 images) covering a consistent 20‐year time‐period prior to the surveys. We repeated our analyses (as described below) with these estimates to assess whether our main findings were robust to the type of burning metric used. As these two sets of analyses differed very little in their findings, we present only those using burn extent estimates from GoogleEarth in the main manuscript but present the alternative results in Appendix S1.

As an index of grazing livestock intensity, adult sheep numbers were estimated in each survey square during the second visit, and assigned to one of four ordinal categories: 0, 1–20, 21–50, and 51+ sheep per km^2^.

### 2.5 Habitat, topographic and geographic variables

The estimated extent of broad habitat types in each survey square was derived from a 25‐m raster version of the UK Land Cover Map 2015 (Rowland et al., 2017). The most extensive habitats in the surveyed squares were: bog (38.7% of the total area surveyed), heather (30.5%), heather grassland (20.9%), and acid grassland (8.4%). Due to the relative similarity of vegetation in the first three of these categories, and the fact that some degraded bog areas may be indistinguishable from upland heathland on vegetation parameters (Rowland et al., 2017), they were combined to form a single “heath habitats” variable. As a proxy for the extent of cover available to predators such as crows, common buzzards, and foxes, in areas close to survey squares, the same dataset was used to determine summed woodland extent in the eight 1‐km squares surrounding each survey square.

Slope and elevation data were calculated for each 1‐km survey square from 30‐m resolution elevation data downloaded from the United States Geological Survey Shuttle Radar Topography Mission (USGS, 2017). Mean elevation was calculated per 1‐km survey square. Slope was calculated for each 30‐m elevation pixel based on the elevation of the surrounding eight pixels. From these latter data, three slope variables were created by calculating the proportion of each square with slopes of less than 2°, 5°, and 10°.

### 2.6 Statistical analyses

Three waders, European golden plover, Eurasian curlew, and common snipe, were recorded on a sufficient number of plots (64, 77, and 45, respectively) to analyze individually. Similarly, three widespread passerines: Eurasian skylark, Eurasian wren, and meadow pipit (recorded in ≥77 survey squares) and red grouse, the target species for management (recorded in 92 survey squares), were sufficiently widespread to be modeled individually in relation to environmental variables. An additional group, comprising large predatory birds that can sometimes predate ground‐nesting waders and grouse (red kite *Milvus milvus*, northern goshawk *Accipiter gentilis*, Eurasian sparrowhawk *Accipiter nisus*, common buzzard *Buteo buteo*, short‐eared owl *Asio flammeus*, peregrine falcon *Falco peregrinus*, and northern raven), was also modeled. These species were chosen to represent (a) species targeted by management to maximize numbers (red grouse), (b) the most frequently recorded ground‐nesting waders that might be affected by management (European golden plover, Eurasian curlew, common snipe), (c) common, widely distributed species that are less closely linked to heather moorland and, so, may be less directly affected by specific elements of management (Eurasian skylark, Eurasian wren, meadow pipit), and (d) a suite of birds that might be expected to respond positively to prey availability (large predatory birds). This latter group was modeled together due to the low number of records for individual species. Generalized linear mixed‐effects models (GLMMs) were fitted for each species/group using the “glmmTMB” function in R (Brooks et al., 2017; R Core Team, 2017). Four types of model were considered, to suit the level of dispersion and zero‐inflation in the abundance data of each species/group: (a) Poisson regression, (b) negative binomial regression, (c) zero‐inflated Poisson regression, and (d) zero‐inflated negative binomial regression. The most appropriate model type was selected for each species following exploration of dispersion and zero‐inflation in their abundance data, and comparisons of model parsimony among maximal models of each type using the Akaike Information Criterion (AIC). Models were fitted with site‐level random intercepts (*n* = 18), to account for nonindependence among survey squares within each estate. Spatial autocorrelation in bird abundance, which might result from this clustering, was tested for using Moran's I statistic. Significant autocorrelation was identified for all species/groups, except for large predatory birds. Models were fitted with all possible combinations of environmental variables as predictors (see Table 2), while not allowing cooccurrence of highly correlated variables (*r* ≥ .70), which included predator control and burning (*r* = .70). A curvilinear relationship between abundance and elevation was tested for by including a quadratic effect of elevation. The abundance of potential avian prey species (the number of individuals of wader species and red grouse) was included as a predictor for large predatory species only. All continuous predictors were standardized ([*x*−*µ*]/*σ*) to produce model coefficients comparable among predictors. For each species, models were selected with the most parsimonious combination of predictors, using AIC. Specifically, we considered models with ΔAIC ≤ 6 and lower than simpler nested models to have support (Richards, 2015) and included these in a top model set for each species. Additionally, Akaike model‐averaged standardized coefficients were calculated across all models for each species to illustrate the strength of evidence for different effects. Predictors occurring within the best performing model, and consistently throughout top model sets, were considered to have strong support.

**Table 2:** Summary of predictor variables used in GLMMs

Variable name	Description	Data source
Burning	Estimated % of survey square under burning management	Google Earth 2003–2016
Elevation	Mean elevation in survey square	Shuttle Radar Topography Mission (USGS, 2017)
Slope (<2°)	Proportion of square with slope <2°	Shuttle Radar Topography Mission (USGS, 2017)
Slope (<5°)	Proportion of square with slope <5°	Shuttle Radar Topography Mission (USGS, 2017)
Slope (<10°)	Proportion of square with slope <10°	Shuttle Radar Topography Mission (USGS, 2017)
Predator control	Full‐time equivalent predator control per 1,000 ha	Interviews with site representatives
Woodland	Woodland cover in the eight 1‐km squares surrounding survey square	CEH Land Cover Map 2015 (Rowland et al., 2017)
Heath habitats	% cover of combined heather, heather grassland and bog	CEH Land Cover Map 2015 (Rowland et al., 2017)
Acid grassland	% cover in survey square	CEH Land Cover Map 2015 (Rowland et al., 2017)
Sheep	Scale 1–4 representing classes: 0, 1–20, 21–50 and >50	Field surveys
Avian prey abundance^a^	Numbers individuals of waders and red grouse	Field surveys

a Avian prey abundance only considered for the large predatory birds model.

Spatial autocorrelation was assessed in the residuals of the best performing model for each species using Moran's I statistic. No models had significant Moran's I statistics, indicating that the random intercept models adequately dealt with any autocorrelation. Collinearity was assessed in the best performing models using variance inflation factors; no models contained predictors with variance inflation factors >3 (Zuur, Ieno, & Elphick, 2010). The overall fit of each best model was evaluated using pseudo *R* ^2^, calculated as the squared correlation coefficient between fitted and observed values.

Next, we examined further the fitted effects of management identified in our models. Firstly, the relative importance of predator control and burning was assessed by comparing the mean and range of Akaike model‐averaged standardized coefficients for each predictor, across all 96 models in which each was present. Secondly, we explored the potential for nonlinear effects of management on bird abundance. Specifically, for species for which predator control was selected in the best model, this GLMM was refitted with a saturating effect of predator control of the form *a*.(1−e^−^ *^b^* ^.x^) (i.e., a positive curvilinear effect that levels off as predator control increases), for which *b* was parameterised using one‐dimensional optimization, implemented using the “optimize” function in R. The parsimony of these GLMMs was compared with that of the original models using AIC, and the best performing models selected (Appendix S2). The fitted effects of predator control on abundance from these models were explored graphically.

## 3 RESULTS

**Figure 2:**
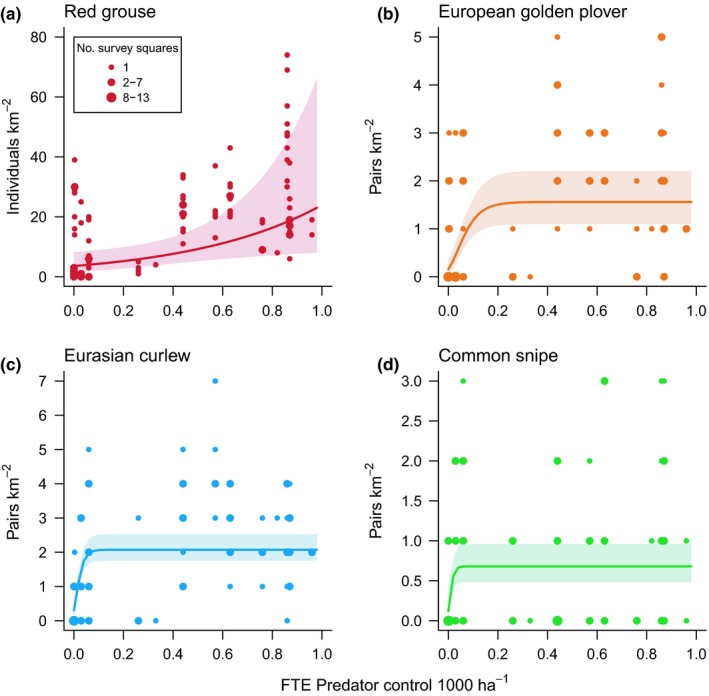
Responses of red grouse and three wader species to predator control intensity. Predator control (expressed here as the number of full‐time equivalent staff carrying out predator control per 1,000 ha) was selected in the best performing model for each of the plotted species. Lines indicate population‐level fitted estimates from the best performing models, with other predictors held at mean values. Shaded areas represent 95% confidence intervals around these estimates. For (a) the best performing model was fitted with a linear effect of predator control, while for (b–d) the best performing models were refitted with saturating nonlinear effects of predator control. Points represent individual 1 km^2^ census areas. Point size indicates the number of survey squares corresponding to each data point

**Figure 3:**
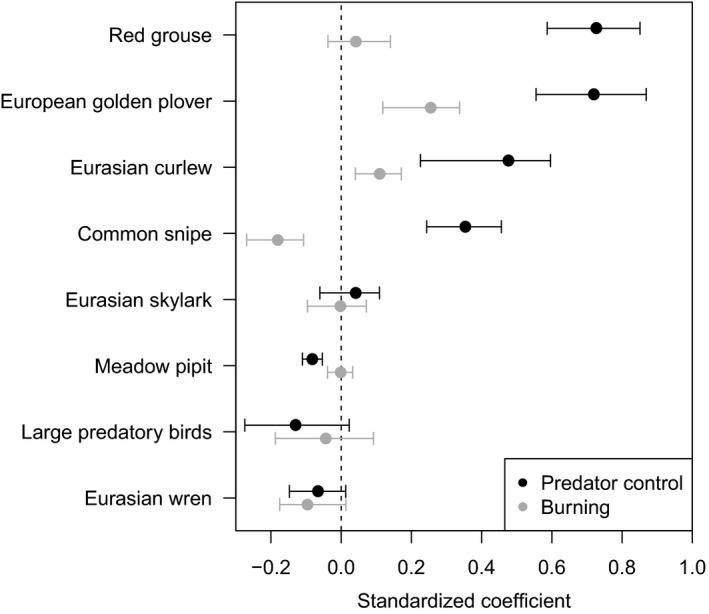
Akaike model‐averaged standardized linear coefficients for the effects of predator control and burning across models containing different combinations of predictors (96 models in each case). As coefficients are standardized their effect sizes can be directly compared. Models containing both predictors together were not considered due to their high collinearity (*r* = .70). Points indicate means and lines indicate ranges

As hypothesized, there was evidence for positive associations between predator control and the abundance of European golden plover, Eurasian curlew, common snipe, and red grouse (Figure 2). Predator control had the strongest model‐averaged coefficient for European golden plover and Eurasian curlew, and the second strongest for common snipe and red grouse (Table 3). The association was most pronounced for red grouse, which displayed increasing numbers with increasing magnitude of predator control effort. The support for this effect was weaker for common snipe, with the null model being selected in the top model set for this species (ΔAIC = 4.99). For the three wader species, the relationships were best described by saturating functions (Table 4), with marked increases in numbers associated with increasing predator control effort up to a point, after which further intensifying of predator control had little effect. The only evidence for an effect of burning for these species was a very weak positive relationship between burning and European golden plover numbers, although burning was not included in the best model for this species (Table 3; Appendix S2). While the relative influences of predator control and burning could not be compared within the same models due to their collinearity, mean coefficients calculated across all models in which each occurred individually indicate much stronger effects of predator control than burning on the abundances of European golden plover, Eurasian curlew, and common snipe and of red grouse (Figure 3). The only evidence for predator control or burning influencing any other species or group was a weak negative effect of burning on Eurasian wren abundance (Figure 3; Table 3).

**Table 3:** Akaike model‐averaged standardized linear coefficients and performance statistics for best models of spatial variation in bird abundance

Species	Red grouse	European golden plover	Eurasian curlew	Common snipe	Meadow pipit	Eurasian wren	Eurasian skylark	Large predatory species
Predator control	**0.51**	**0.60**	**0.40**	**0.18**	−0.02	−0.01	0.01	−0.05
Burning	0.00	**0.05**	0.00	−0.03	0.00	**−0.06**	0.00	0.00
Sheep					**+**			
Heath	**0.07**	0.01	0.00	−0.04	0.00	**0.06**	**−0.05**	0.04
Acid grassland	**−0.06**	0.00	0.00	0.03	0.00	**−0.05**	**0.05**	−0.04
Woodland	−0.07	**−0.29**	**−0.33**	**0.05**	0.02	0.00	−0.01	**0.31**
Elevation	**1.22**	**0.17**	−0.02	**−0.30**	**−0.26**	**−0.59**	−0.12	**−0.02**
Elevation^2^	**−1.42**	0.23	0.00	−0.09	0.06	0.10	0.05	−0.09
Slope	**−0.08 (<5˚)**	−0.03 (<5˚)	**0.06 (<5˚)**	**−0.05 (<5˚)**	**−0.02 (<10˚)**	**−0.33 (<2˚)**	**0.04 (<10˚)**	−0.04 (<5˚)
Avian prey abundance	—	—	—	—	—	—	—	0.13
Distribution family	Neg. binomial	Poisson	Poisson	Poisson	Neg. binomial	Poisson	Neg. binomial	Poisson
Zero‐inflated		✓	✓					✓
Best model *R* ^2^	.87	.52	.44	.18	.67	.34	.66	.29
Null model in top set				✓			✓	✓
Null model ΔAIC	28.77	10.36	9.07	4.99	16.24	43.42	0.68	3.66

Note

Model‐averaged coefficients were calculated across all models for each species. Coefficients highlighted in bold indicate predictors selected in the top model set of a given species. All models were fitted with site‐level random intercepts. For the slope variable, numbers in parentheses indicate the best performing threshold for this predictor. An effect of avian prey abundance was only included in models of large predatory species.

Abbreviation: AIC, Akaike Information Criterion.

**Table 4:** Relative performance of models fitted with linear (*a*.*x*) and saturating effects [*a*.(1−e^−^ *^b^* ^.^ *^x^*)] of predator control on the abundance of four selected species. There was evidence for a saturating effect for the three wader species, but not red grouse

Effect of predator control (*x*)	Red grouse		Eurasian curlew		European golden plover		Common snipe	
	LL	ΔAIC	LL	ΔAIC	LL	ΔAIC	LL	ΔAIC
*a*.*x*	−322.95	0.00	−153.00	9.79	−127.45	5.42	−103.25	3.51
*a*.(1−e^−^ *^b^* ^.^ *^x^*)	−322.83	1.75	−147.11	0.00	−123.74	0.00	−100.50	0.00

Abbreviation: AIC, Akaike Information Criterion.

Vegetation cover and topography also played important roles in the abundance of most species. For example, surrounding woodland extent negatively influenced the abundances of European golden plover and Eurasian curlew but was associated with a higher abundance of large predatory birds (though the null model occurred in the top model set for this group; Table 3). Eurasian wren responded positively to heath extent. Elevation occurred in the best performing models of five species, with numbers of common snipe, Eurasian wren and meadow pipit being higher at lower sites, and European golden plover being more numerous at higher sites. A quadratic effect was selected for red grouse, suggesting higher grouse numbers at intermediate elevations. An effect of slope was selected in the best model of six species. Eurasian wren abundance was negatively associated with the spatial extent of flat areas, while effects for other species were relatively weak. The null model occurred within the top model sets for common snipe, Eurasian skylark and large predatory birds, while the pseudo *R* ^2^ of the common snipe and large predatory bird models were quite low (.18 and .29, respectively). Thus, our findings for these species were less well supported than were those for red grouse (pseudo *R* ^2^, .87), European golden plover (pseudo *R* ^2^, .52), Eurasian curlew (pseudo *R* ^2^, .44), Eurasian wren (pseudo *R* ^2^, .34), and meadow pipit (pseudo *R* ^2^, .67).

We did not detect any support for an association between numbers of large predatory birds and their avian prey. While there was a moderately high model‐averaged coefficient for avian prey abundance, this effect was not selected in the top model set.

## 4 DISCUSSION

We illustrate that population densities of three wader species—European golden plover, Eurasian curlew, and common snipe—were, along with red grouse, positively influenced by management associated with driven grouse shooting, more specifically predator control (Figure 2). Interestingly, our analyses indicate that the abundance‐predator control relationships for these waders saturate at relatively low levels of predator control, suggesting that there are diminishing benefits of increasing predator control for these species. In contrast, we found little support for a strong influence of burning on upland bird species. This evidence, which helps to clarify the relative importance of these management actions, has potential to underpin scenario‐based predictive models and field experiments to evaluate how these species would be affected by future changes in upland management.

Our study is an example of how applied ecology can inform a debate surrounding entrenched conservation conflicts. For example, our models suggest that both grouse and ground‐breeding wader numbers would be less impacted by cessation or reduction in burning compared with the complete removal of predator control, which would likely adversely impact all such species. By contrast, a moderate reduction in the intensity of predator control might not markedly affect ground‐nesting wader numbers but would be likely to impact red grouse populations. Such information could usefully inform the debate over alternative management scenarios for the uplands, especially those debates that seek to satisfy multiple objectives (e.g., recreational shooting, carbon sequestration, and key biodiversity conservation). Our findings, from a single—but extensive—snapshot survey, also provide justification for further, detailed experimental studies of the impacts of varying the intensity of the two major management strategies (burning and predator control) on UK moorlands.

While a range of species benefited from management for driven grouse shooting on our study sites, we found poor support for any positive influence of burning. Burning and predator control are closely linked activities in intensive management for driven grouse shooting and, unsurprisingly, the intensity of these activities was correlated in our data (*r* = .70). However, by fitting models with each action independently, it was possible to compare the relative strength of evidence for these activities and their effect sizes (Figure 3). It is still likely, however, that burning does play a role in shaping bird assemblages independently of predator control (e.g., Robertson, Newborn, Richardson, & Baines, 2017; Whittingham, Percival, & Brown, 2002). Experimental studies teasing apart these associations are generally restricted to single sites with, for example, Douglas et al. (2017) finding Eurasian golden plover abundance to increase on experimentally burned areas. Teasing apart these associations over a regional scale is challenging, due to the difficulty in finding field sites with sufficiently varying magnitudes of burning and predator control. Nonetheless, our results suggest that the importance of burning is considerably lower than that of predator control for upland waders.

For the other study species—Eurasian skylark, Eurasian wren, meadow pipit, and large predatory species—the only identified association with moorland management was a weak negative association between burning and Eurasian wren abundance. This could be linked to a reduction in overall vegetation height and structure, caused by burning, though analysis using remotely sensed data (Appendix S1) found no effect at all, so this relationship should be treated with caution. Alternatively, the relationship could be linked to Eurasian wren preferring sloping ground (Table 3) which tends to be less‐frequently burned. The most abundant bird encountered, meadow pipit, favors habitat mosaics containing acid grassland, in addition to heather (Pearce‐Higgins & Grant, 2006; Vanhinsbergh & Chamberlain, 2001), and, within our largely heather‐dominated survey squares, the distribution of such mixes may be independent of moorland management intensity. Previous studies have shown a negative association of meadow pipits with areas of grouse moor (Newey et al., 2016; Smith, Redpath, Campbell, & Thirgood, 2001; Tharme et al., 2001). Our findings did not identify specific evidence of grouse moor management practices on their abundance. We did identify a positive association of sheep numbers with meadow pipit abundance. This may reflect sheep aggregating on areas with a higher availability of grazing resources, though acid grassland extent, itself, was not associated with increased pipit numbers.

The abundance of large predatory birds was not related to legal predator control or burning and did not respond to the higher availability of grouse and waders as prey items on intensively managed moors. The models for these species performed poorly, with the null model featuring in the best model set. Predatory birds have been shown to exhibit a range of numerical and functional responses to grouse moor management and, for several studies, it has been demonstrated that their abundance can be positively associated with grouse abundance and legal predator control (Baines et al., 2008; Ludwig, Roos, Bubb, & Baines, 2017). The low numbers of large predatory birds across many of our study sites (mean per survey square = 0.80 birds, *SD* = 1.14) could reflect wider population suppression arising from illegal persecution of birds of prey on grouse moors (e.g., Amar et al., 2012; Murgatroyd et al., 2019; Whitfield & Fielding, 2017), and more widely. Thus, the poor performance of our models could relate to our lack of national data on intensity of illegal persecution. However, our need to combine these species into a single category, due to their low numbers, may have obscured effects for individual species. For example, populations of some species may be more sensitive to availability of nonavian prey (e.g., Francksen, Whittingham, Ludwig, Roos, & Baines, 2017) and, thus, may be independent of grouse and wader abundance.

### 4.1 Implications for moorland management

There is intense debate about how the UK's uplands should be managed. Part of this debate focusses on grouse moor management but other considerations, such as the carbon sequestration potential of UK uplands, are increasing likely to influence future management. Recent attention has concentrated on the wider impacts of driven grouse shooting, including on factors such as carbon storage, water quality, and flood alleviation (e.g., Sotherton et al., 2017; Thompson et al., 2016) and on species of conservation concern (Watson & Wilson, 2018) as well as on the incompatibility of raptor protection with economic viability of driven grouse shooting (Sotherton et al., 2009; Thompson et al., 2009). Petitions supporting and opposing driven grouse shooting culminated in a UK parliamentary debate on the topic (Anon, 2016), while, with the increased use of satellite tagging of raptors, cases of unusual disappearances of tagged individuals in the uplands continue to occur over areas of moorland managed for driven grouse shooting (e.g., Murgatroyd et al., 2019; Whitfield & Fielding, 2017), further dividing the shooting and conservation communities.

Our study provides an important evidence‐base for developing experimental systems and predictive models of how changes in the management of upland areas might influence the UK populations of upland bird species. There is a range of outcomes that could affect moorlands if driven grouse shooting were to cease, or management associated with it were severely curtailed. Firstly, legal predator control would be unlikely to continue at current levels. As such, given the magnitude of parameter estimates that we calculated for the effect of predator control on moorland breeding populations of European golden plover and Eurasian curlew in particular, as well as red grouse, it is likely that population densities of these species would be reduced in upland areas that are currently managed intensively if predator control were to cease. However, some localized predator control may still be carried out by tenant graziers for sheep protection or, toward moorland edges, control may be continued at some sites by pheasant shoots. Although we identified saturating effects of predator control on abundance of European golden plover and Eurasian curlew, it remains unclear if low or moderate levels would be sufficient to maintain their populations. Nonetheless, considering recent evidence for the declining conservation status of the Eurasian curlew (Brown et al., 2015), stabilizing or reversing declines in their populations might not be possible without channeling high levels of resources into predator control specifically for species conservation purposes, alongside a range of other measures (as discussed by Franks et al., 2017).

Heather burning may also largely cease in an absence of driven grouse shooting, or its intensity and extent may be curtailed through regulatory controls. Some, though, may be carried out to improve conditions for browsing by sheep (albeit on a wider patch scale than that for grouse management) or, through agri‐environment incentives, for maintaining habitat quality. Currently, burning in the UK uplands appears to be increasing in extent and concern is growing that this may increase carbon loss and reduce flood mitigation properties of moorlands (Douglas et al., 2015). Heather moorland is an anthropogenic habitat, maintained largely by burning, sometimes in conjunction with grazing by sheep, cattle, or deer. A decline in such management may lead to an increase in the proportion of grasses and, where climate and grazing allow, establishment of scrub or woodland (e.g., Gimingham, 1989). Our models suggest that European golden plover and Eurasian curlew would decline if woodland cover increased, including where the woodland increase is on adjacent areas in addition to directly on sites occupied by these waders (Table 3).

Our study found no significant link between management associated with driven grouse shooting and the numbers of larger predatory birds encountered. This was contrary to our hypothesis that numbers of noncontrolled predators that target ground‐nesting birds should occur at greater densities on moors where other predators are legally controlled and abundances of prey species are higher (Table 3). Some evidence suggests that abandoning management associated with driven grouse shooting may be detrimental to populations of some predatory bird species, due to consequential habitat changes and increases in mammalian predators (e.g., Baines et al., 2008). On the other hand, cessation of driven grouse shooting could lead to reductions in the illegal killing of several raptor species in upland areas (e.g., Amar et al., 2012; Murgatroyd et al., 2019; Whitfield & Fielding, 2017). Such illegal activity could be a factor in our failure to identify any association of grouse moor management with our sightings of larger predatory birds. This could also explain why our predictive models for such species were relatively weak.

## 5 CONCLUSIONS

An important issue in the debate over driven grouse shooting is concern for the wider assemblage of nontarget moorland bird species. Our research clearly demonstrates that management associated with driven grouse shooting, in particular predator control, benefits ground‐nesting waders, as well as red grouse, when compared to areas of similar habitat with little or no predator control. While cessation of driven grouse shooting and its associated management activities would seem likely to impact these species negatively, the retention of low or moderate levels of predator control could potentially still benefit them due to the functional forms of the abundance‐predator control relationships. These results add to our understanding of the likely consequences of different management options for moorland bird species of conservation concern, providing the evidence‐base to inform scenario‐based predictive population models. As the debate surrounding the issue progresses, it is vital that a strong evidence‐base is used in decision‐making and policy formation and that the implications of changes in moorland practices, or continuation of present management, are carefully considered.

## CONFLICT OF INTEREST

None declared.

## AUTHOR CONTRIBUTIONS

MJW and SGW conceived the idea and, together with NAL, designed methodology; Fieldwork was carried out by NAL, MH, and RJ; NAL and THEM analyzed the data; NAL and THEM led the writing of the manuscript. All authors contributed critically to the drafts and gave final approval for publication. We thank Chris Elphick and three anonymous referees for useful comments on drafts of the manuscript.

## Data Availability

Data are archived in the Dryad digital repository (https://doi.org/10.5061/dryad.cv1gb10).
