# Correction to “The Influence of Different Aspects of Grouse Moorland Management on Nontarget Bird Assemblages”

**DOI:** 10.1002/ece3.74434

**Published:** 2026-10-07

N. A. Littlewood , T. H. E. Mason , M. Hughes , R. Jaques , M. J. Whittingham , and S. G. Willis . 2019. “The Influence of Different Aspects of Grouse Moorland Management on Nontarget Bird Assemblages.” Ecology and Evolution 9, no. 19: 11089–11101. 10.1002/ece3.5613.PMC680203531641457

A third party has reported that the research covered by this article received funding from owners of land managed for sport shooting of Red Grouse. The evidence provided by the third party (a newspaper article from August 2017) exists in the public record and was available prior to the publication of this article. The Charities Aid Foundation, Newcastle University and the Durham University all confirm that due diligence was carried out on the organization making the donation and further confirm that the authors were not aware of the identity of the anonymous donor(s) prior to the publication of this article. However, in interests of clarity, the authors have chosen to publish a correction to include the following Conflict of Interest statement:

**CONFLICT OF INTEREST**

This work was funded by an anonymous donation via the Southampton Row Trust Ltd., the Charities Aid Foundation American Donor Fund (CAF). A UK newspaper reported that this donation was funded by owners of land managed for sport shooting of Red Grouse. In accordance with the policies of the CAF, the name(s) of donor(s) were not disclosed to the universities or to the authors. The CAF, Newcastle University and Durham University all carried out due diligence investigations on the organization making the donation and each confirmed that the identity of the donor(s) has not been disclosed to the authors. The authors remain unaware of the specific identity of the funder(s) of this research. In accordance with the gift agreement, as stipulated by Newcastle University and Durham University, the results and conclusions presented in the article were not influenced by donors.

