# Supplementary material for: The influence of different aspects of grouse moorland management on nontarget bird assemblages
Source: Ecol Evol. 2019 Sep 26;9(19):11089–101. doi: 10.1002/ece3.5613 (PMC6802035; doi:10.1002/ece3.5613)
Supplement: Supplementary file 1 [file ECE3-9-11089-s001.docx]

Appendix 1. Methods and results for supplementary analysis using a remotely sensed estimate of burning.

We calculated an alternative index of burning extent from remote sensing data and refitted our models using these estimates to assess the robustness of our findings to how burning was quantified. We estimated burning from Landsat 5 and 7 images for the study area spanning the 20 years prior to our surveys (i.e., matching the time period of our visually inferred estimates; Yallop et al., 2006). Landsat 30-m scale images were collated from May of each year, after the period of legal prescribed burning of heather in the UK (October-April). Of the images taken in May, the image with the lowest cloud cover in each year was selected. The difference normalised burn ratio (dNBR) was calculated between images from adjacent years (López García, 1991). This index measures how surface reflectance changes between adjacent time-periods and can determine when healthy vegetation is replaced by burned vegetation. We used dNBR to calculate the total area of each survey square that had been burned at least once over the 20-year period. We tested different thresholds for classifying burnt vs unburnt areas by calculating remotely sensed burning extent using a range of thresholds, spanning those recommended by the US Geological Survey (USGS; <https://www.frames.gov/documents/projects/firemon/FIREMON_LandscapeAssessment.pdf>). We then correlated these with our visually inferred indices estimated from Google Earth images. We selected the threshold that maximised Pearson’s correlation coefficients both across survey squares and mean levels on estates (*r*: survey square, 0.34; estate, 0.71). Our selected threshold (0.50) coincides with USGS-recommended thresholds for moderate severity burns. We refitted models with remotely sensed burning estimates in place of visually inferred estimates and performed model selection, as described in the Methods (Table A1). As in the main analysis, we compared the mean and range of coefficients for burning and predator control (Fig. A1).

Table A1. Akaike model-averaged standardised linear coefficients and performance statistics for best models of spatial variation in bird abundance using burning extent estimated from remote sensing data. Model-averaged coefficients were calculated across the top model sets for each species. Coefficients highlighted in bold indicate predictors selected in the top model set of a given species. All models were fitted with site-level random intercepts. For the slope variable, numbers in parentheses indicate the best performing threshold for this predictor. An effect of avian prey abundance was only included in models of large predatory species.

| **Species** | Red grouse | European golden plover | Eurasian curlew | Common snipe | Meadow pipit | Eurasian wren | Eurasian skylark | Large predatory species |
| --- | --- | --- | --- | --- | --- | --- | --- | --- |
| **Predator control** | **0.51** | **0.59** | **0.40** | **0.17** | -0.03 | -0.02 | 0.01 | -0.05 |
| **Burning** | 0.00 | 0.05 | -0.01 | -0.05 | -0.01 | 0.01 | -0.06 | 0.01 |
| **Sheep** |  |  |  |  | **+** |  |  |  |
| **Heath** | **0.07** | 0.15 | 0.00 | -0.04 | 0.00 | **0.05** | **-0.05** | 0.04 |
| **Acid grassland** | **-0.06** | -0.01 | 0.00 | 0.03 | 0.00 | **-0.05** | **0.05** | -0.04 |
| **Woodland** | -0.07 | **-0.19** | **-0.33** | **0.06** | 0.03 | 0.01 | 0.00 | **0.30** |
| **Elevation** | **1.22** | **0.18** | 0.00 | **-0.39** | **-0.27** | **-0.61** | -0.15 | **-0.02** |
| **Elevation^2^** | **-1.42** | 0.22 | 0.00 | 0.02 | 0.08 | 0.13 | 0.06 | -0.10 |
| **Slope** | **-0.08 (<5˚)** | 0.00 (<5˚) | **0.06 (<5˚)** | **-0.05 (<5˚)** | **-0.02 (<10˚)** | **-0.25 (<5˚)** | **0.04 (<10˚)** | **0.04 (<5˚)** |
| **Avian prey abundance** | - | - | - | - | - | - | - | 0.13 |
| **Distribution family** | Neg. binomial | Poisson | Poisson | Poisson | Neg. binomial | Poisson | Neg. binomial | Poisson |
| **Zero-inflated** |  | ✓ | ✓ |  |  |  |  | ✓ |
| **Best model *R*^2^** | 0.87 | 0.52 | 0.44 | 0.18 | 0.67 | 0.37 | 0.66 | 0.29 |
| **Null model in top set** |  |  |  | ✓ |  |  | ✓ | ✓ |
| **Null model ΔAIC** | 28.77 | 10.36 | 9.07 | 4.99 | 16.24 | 41.82 | 0.68 | 3.66 |


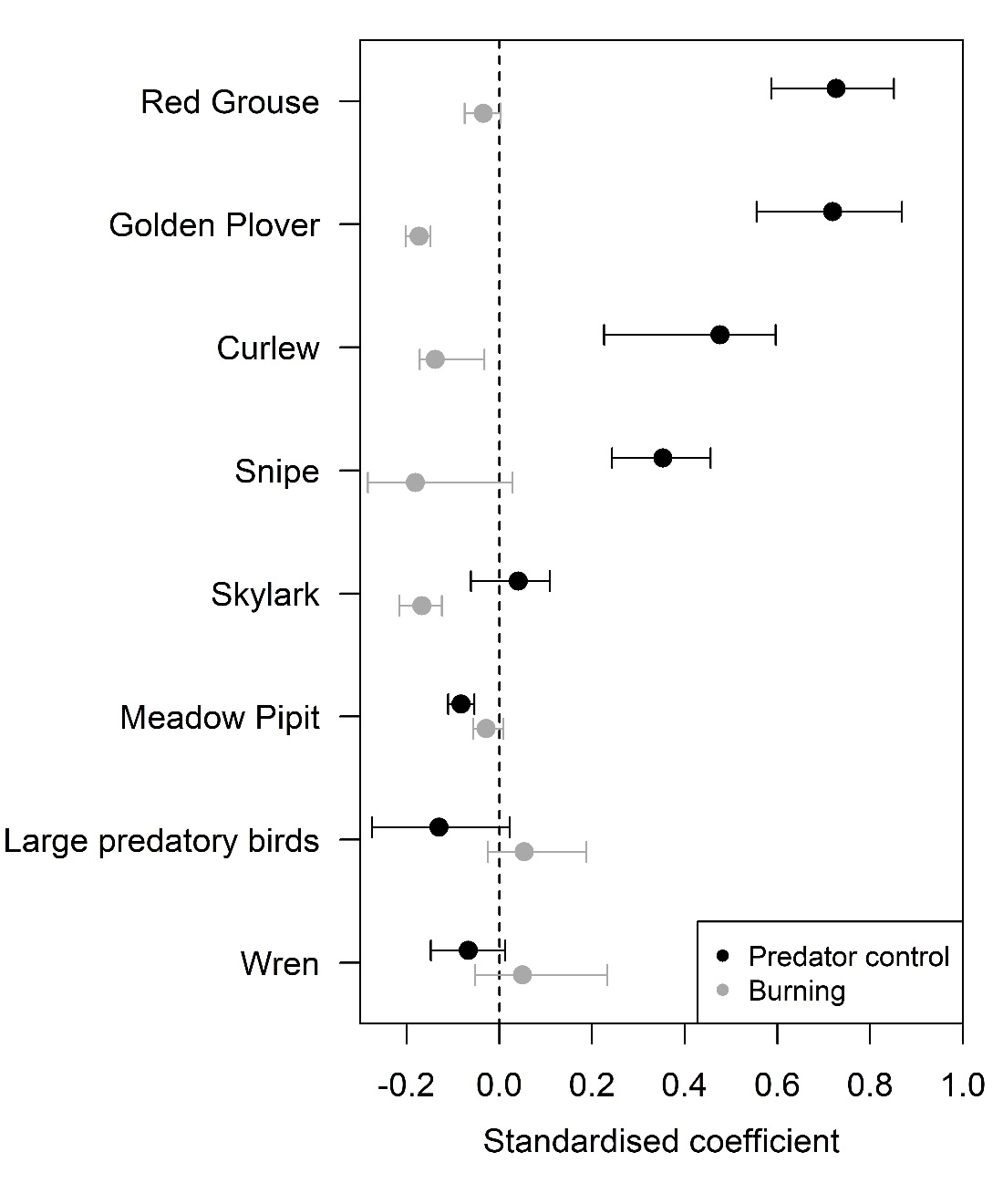


Figure A1. Akaike model-averaged standardised linear coefficients (i.e. scaled so effect sizes can be directly compared) for the effects of predator control and remotely sensed burning across models containing different combinations of predictors (96 models in each case). As coefficients are standardised their effect sizes can be directly compared. Points indicate means and lines indicate ranges.

References

López García, M.J. & Caselles, V. (1991) Mapping burns and natural reforestation using Thematic Mapper data. *Geocarto International* 6: 31–37.

Yallop, A.R., Thacker, J.I., Thomas, G., Stephens, M., Clutterbuck, B., Brewer, T. & Sannier, C.A.D. (2006) The extent and intensity of management burning in the English upland. *Journal of Applied Ecology* 43: 1138–1148.

Appendix 2: Linear coefficients from top sets of models explaining spatial variation in bird abundance. All models were fitted with site-level random intercepts.

1. **Red Grouse**

| **Pred. control** | **Heath** | **Grass** | **Elev.** | **Elev.^2^** | **Slope < 2°** | **Slope < 5°** | **Slope < 10°** | ***K*** | **LL** | **ΔAIC** |
| --- | --- | --- | --- | --- | --- | --- | --- | --- | --- | --- |
| 0.68 | 0.14 |  | 1.18 | -1.39 |  | -0.16 |  | 8 | -322.95 | 0.00 |
| 0.68 |  | -0.13 | 1.21 | -1.42 |  | -0.16 |  | 8 | -323.07 | 0.22 |
| 0.66 | 0.16 |  | 1.31 | -1.49 |  |  | -0.15 | 8 | -323.65 | 1.39 |
| 0.66 |  | -0.15 | 1.35 | -1.52 |  |  | -0.15 | 8 | -323.73 | 1.55 |
| 0.70 | 0.10 |  | 1.06 | -1.27 | -0.14 |  |  | 8 | -324.00 | 2.10 |
| 0.71 |  | -0.09 | 1.08 | -1.29 | -0.14 |  |  | 8 | -324.18 | 2.46 |
| 0.72 |  |  | 1.17 | -1.35 | -0.13 |  |  | 7 | -325.51 | 3.11 |
| 0.71 |  |  | 1.28 | -1.46 |  | -0.13 |  | 7 | -325.56 | 3.22 |
|  | 0.14 |  | 1.22 | -1.45 |  | -0.16 |  | 7 | -325.59 | 3.28 |
|  |  | -0.13 | 1.25 | -1.47 |  | -0.17 |  | 7 | -325.72 | 3.53 |
|  | 0.16 |  | 1.36 | -1.55 |  |  | -0.15 | 7 | -326.23 | 4.55 |
|  |  | -0.15 | 1.40 | -1.59 |  |  | -0.16 | 7 | -326.31 | 4.72 |
| 0.70 |  |  | 1.35 | -1.51 |  |  | -0.10 | 7 | -326.46 | 5.01 |
|  | 0.11 |  | 1.09 | -1.32 | -0.15 |  |  | 7 | -326.73 | 5.56 |
|  |  | -0.09 | 1.12 | -1.34 | -0.15 |  |  | 7 | -326.93 | 5.94 |

1. **European golden plover**

| **Predator control** | **Burning** | **Woodland** | **Elevation** | ***K*** | **LL** | **ΔAIC** |
| --- | --- | --- | --- | --- | --- | --- |
| 0.67 |  | -0.37 | 0.38 | 6 | -127.45 | 0.00 |
| 0.79 |  |  | 0.45 | 5 | -128.56 | 0.22 |
|  | 0.32 |  | 0.55 | 5 | -129.78 | 2.67 |
| 0.58 |  | -0.54 |  | 5 | -130.35 | 3.81 |

1. **Eurasian curlew**

| **Predator control** | **Woodland** | **Slope < 2˚** | **Slope < 5˚** | ***K*** | **LL** | **ΔAIC** |
| --- | --- | --- | --- | --- | --- | --- |
| 0.44 | -0.39 |  | 0.15 | 6 | -153.00 | 0.00 |
| 0.43 | -0.41 | 0.13 |  | 6 | -153.04 | 0.08 |
| 0.45 | -0.35 |  |  | 5 | -154.34 | 0.68 |
| 0.56 |  |  |  | 4 | -156.71 | 3.42 |
|  | -0.39 |  |  | 4 | -157.90 | 5.81 |

1. **Common snipe**

| **Predator control** | **Heath habitats** | **Woodland** | **Elevation** | **Slope < 5˚** | ***K*** | **LL** | **ΔAIC** |
| --- | --- | --- | --- | --- | --- | --- | --- |
| 0.29 |  |  | -0.52 | -0.20 | 5 | -103.25 | 0.00 |
| 0.29 |  |  | -0.46 |  | 4 | -104.28 | 0.06 |
|  |  |  | -0.45 |  | 3 | -105.53 | 0.57 |
| 0.36 |  | 0.31 |  |  | 4 | -106.27 | 4.04 |
| 0.33 | -0.23 |  |  |  | 4 | -106.28 | 4.06 |
|  |  | 0.25 |  |  | 3 | -107.73 | 4.95 |
|  |  |  |  |  | 2 | -108.75 | 4.99 |

1. **Meadow Pipit**

| **Sheep** | **Elevation** | **Slope < 5˚** | **Slope < 10˚** | ***K*** | **LL** | **ΔAIC** |
| --- | --- | --- | --- | --- | --- | --- |
| + | -0.21 |  | -0.06 | 8 | -474.23 | 0.00 |
| + | -0.21 | -0.05 |  | 8 | -474.71 | 0.95 |
| + | -0.22 |  |  | 7 | -475.87 | 1.27 |
|  | -0.20 |  | -0.08 | 5 | -479.06 | 3.65 |
|  | -0.22 | -0.08 |  | 5 | -479.66 | 4.87 |

1. **Eurasian wren**

| **Burning** | **Heath habitats** | **Acid grassland** | **Elevation** | **Slope < 2˚** | **Slope < 5˚** | ***K*** | **LL** | **ΔAIC** |
| --- | --- | --- | --- | --- | --- | --- | --- | --- |
| -0.17 | 0.15 |  | -0.52 | -0.58 |  | 6 | -157.90 | 0.00 |
| -0.16 |  | -0.14 | -0.50 | -0.59 |  | 6 | -158.06 | 0.32 |
|  |  | -0.14 | -0.50 |  | -0.49 | 5 | -159.70 | 1.61 |
|  | 0.13 |  | -0.52 |  | -0.48 | 5 | -159.81 | 1.83 |
| -0.12 |  |  | -0.46 | -0.53 |  | 5 | -159.85 | 1.90 |
|  |  | -0.10 | -0.50 | -0.58 |  | 5 | -160.11 | 2.43 |
|  |  |  | -0.47 | -0.53 |  | 4 | -161.13 | 2.46 |
|  |  |  | -0.47 |  | -0.43 | 4 | -161.30 | 2.81 |
|  |  |  |  |  |  |  |  |  |

1. **Eurasian skylark**

| **Heath habitats** | **Acid grassland** | **Slope < 10˚** | ***K*** | **LL** | **ΔAIC** |
| --- | --- | --- | --- | --- | --- |
|  | 0.14 | 0.14 | 5 | -332.57 | 0.00 |
| -0.12 |  |  | 4 | -333.62 | 0.10 |
|  | 0.12 |  | 4 | -333.62 | 0.10 |
|  |  |  | 3 | -334.91 | 0.68 |

1. **Large predatory species**

| **Woodland** | **Elevation** | ***K*** | **LL** | **ΔAIC** |
| --- | --- | --- | --- | --- |
| 0.36 |  | 4 | -121.93 | 0.00 |
|  | -0.29 | 4 | -123.74 | 3.61 |
|  |  | 3 | -124.76 | 3.66 |
